# Complex Spatial Illumination Scheme Optimization of Backscattering Mueller Matrix Polarimetry for Tissue Imaging and Biosensing

**DOI:** 10.3390/bios14040208

**Published:** 2024-04-22

**Authors:** Wei Jiao, Zheng Zhang, Nan Zeng, Rui Hao, Honghui He, Chao He, Hui Ma

**Affiliations:** 1 Guangdong Research Center of Polarization Imaging and Measurement Engineering Technology, Shenzhen Key Laboratory for Minimal Invasive Medical Technologies, Institute of Biopharmaceutical and Health Engineering, Tsinghua Shenzhen International Graduate School, Tsinghua University, Shenzhen 518055, China; jiaow23@mails.tsinghua.edu.cn (W.J.); zhangzhe21@mails.tsinghua.edu.cn (Z.Z.); zengnan@sz.tsinghua.edu.cn (N.Z.); haor22@mails.tsinghua.edu.cn (R.H.); mahui@tsinghua.edu.cn (H.M.); 2 Department of Engineering Science, University of Oxford, Parks Road, Oxford OX1 3PJ, UK

**Keywords:** polarimetry, Mueller matrix, backscattering imaging, spatial illumination, bio-imaging and sensing

## Abstract

Polarization imaging and sensing techniques have shown great potential for biomedical and clinical applications. As a novel optical biosensing technology, Mueller matrix polarimetry can provide abundant microstructural information of tissue samples. However, polarimetric aberrations, which lead to inaccurate characterization of polarization properties, can be induced by uneven biomedical sample surfaces while measuring Mueller matrices with complex spatial illuminations. In this study, we analyze the detailed features of complex spatial illumination-induced aberrations by measuring the backscattering Mueller matrices of experimental phantom and tissue samples. We obtain the aberrations under different spatial illumination schemes in Mueller matrix imaging. Furthermore, we give the corresponding suggestions for selecting appropriate illumination schemes to extract specific polarization properties, and then provide strategies to alleviate polarimetric aberrations by adjusting the incident and detection angles in Mueller matrix imaging. The optimized scheme gives critical criteria for the spatial illumination scheme selection of non-collinear backscattering Mueller matrix measurements, which can be helpful for the further development of quantitative tissue polarimetric imaging and biosensing.

## 1. Introduction

Polarization techniques have been widely applied to biomedical imaging and sensing with their label-free, non-invasive, and microstructure-sensitive advantages [1,2,3]. Mueller matrix (MM) polarimetry is more and more prevalently used nowadays, as it encodes numerous polarization properties comprehensively [4,5,6]. To quantitatively characterize the polarization properties such as diattenuation, retardance, and depolarization, polarization basic parameters (PBPs) are conventionally obtained using Mueller Matrix Polar Decomposition [7], Mueller Matrix Transformation [2], and other MM decomposition methods [8,9,10]. PBPs can increase the image contrast of specific tissue structures with characteristic properties, such as size, shape, fiber orientation, and alignment, thus enhancing polarimetric biosensing [2]. The primary techniques for MM measurement can be categorized as transmission MM microscopy [11] and backscattering MM polarimetry [12,13]. Thin tissue sample (with a thickness less than 3 × 10^1^ μm) measurements can be taken using transmission microscopy, while bulk tissue sample (with a thickness larger than 3 × 10^3^ μm) measurements require backscattering polarimetry, such as bio-structural and optical properties sensing [14,15], polarimetric endoscopy [16,17], minimally invasive surgery [5], and skin tissue analysis [18,19,20] for in vivo scenarios. Despite the fact that backscattering MM polarimetry has great potential for various applications, there are still largely unexplored questions. The precision of MM polarimetry can be affected by many factors, including the low signal-to-noise ratio induced by the light field [21], the azimuthal dependence of the data obtained [22], and other measurement factors [2]. One of these is the uncertain incidence and detection angles resulting from uneven biomedical tissue surfaces, namely the surfaces of internal organs with complicated topography [13].

In general, the backscattering MM polarimetric setup consists of a polarization state generator (PSG) and a polarization state analyzer (PSA), which are located on the same side of the sample. The angle formed between the PSG (or PSA) and the normal of the tissue surface is named incidence angle *θ* (or detection angle *ζ*), and the angle formed between the PSG and PSA is named absolute spatial angle *ψ*, which is the sum of *θ* and *ζ*. There are four backscattering polarimetry schemes, including the following: (i) normal incidence with normal emergent light; (ii) oblique incidence with normal emergent light; (iii) normal incidence with oblique emergent light; (iv) oblique incidence with oblique emergent light. The complex spatial illumination scheme refers to the spatial relationship between the PSG, PSA, and sample, including the four different schemes above. Ideal collinear reflection MM measurement, as in scheme (i), can reduce the amount of measuring errors by avoiding the orientation effects [23]. However, it requires a non-polarizing beam splitter to ensure the emergent light is colinear with incidence, which increases the cost of the device, induces cumulative errors after frequent use, and asks for an intricate calibration [2]. On the other hand, uneven biomedical tissue surfaces make it extremely difficult to take this way. Conversely, the non-linear reflection approach, although inducing orientation effects, can effectively overcome the above problems [13]. Therefore, non-colinear reflection MM measurement equipment has been increasingly used in biomedical studies and applications.

As an optical method, backscattering polarimetry can be used to detect chemical compounds in tissues or cells by optical signals as part of a biosensor, together with other techniques such as surface plasmon resonance [24,25]. For instance, biosensors based on Mueller matrix measurements have been applied to the detection of miRNA [24], bovine serum albumin [25], dengue virus, and glucose [26,27,28]. Specifically, a decomposition Mueller matrix polarimetry was proposed for detecting miRNA [24], showing that the polarization parameter could be used as a quantitative sensing index of chemical compounds. A cutting-edge biosensing method that tracks the binding reaction between bovine serum albumin and its antibodies by measuring the phase difference between p- and s-polarization was demonstrated [25]. Moreover, Mueller matrix polarimetric techniques were also used to sense the glucose concentration in aqueous solutions or detect dengue virus [26,27,28]. However, in the above-mentioned works, we can hardly find a clear criterion of the spatial illumination angle selection, which may have a potential influence on the polarimetric measurement. Both the incidence and detection angles of non-collinear backscattering polarimetric devices are inconsistent in different Mueller matrix polarimetry-based biosensors. For instance, the detection angle was 80° in [24], and the absolute space angle was 60° in [26]. Therefore, we believe that analyzing the impact of complex spatial illumination on backscattering Mueller matrix polarimetry is crucial, which is the focus of this study.

Some recent works have demonstrated that non-colinear reflection can have a complex impact on MM measurement [2]. For instance, the polarimetric aberrations in the MM elements and PBPs at different *θ*, as in scheme (ii), have been analyzed previously [13]. In this study, we analyze the detailed features of complex spatial illumination-induced aberrations for backscattering MM. We measure the MMs of an anisotropic silk phantom designed previously [13] and porcine liver tissue to explore the influence of the different schemes (ii)−(iv), on MM imaging. The experimental results demonstrate that different polarimetric aberrations can be induced in two-periodic MM elements, as in schemes (ii) and (iii), which are verified using the mean-square error (MSE) and energy spectral density. Furthermore, we find that when measuring MMs with an adjusting distribution of *θ* and *ζ*, as in scheme (iv), the aberrations can be significantly alleviated. Additionally, as *ζ* increases, obvious image distortions can be observed, performing as transverse compressions parallel to the incident plane. The relationship between *ζ* and the extent of distortions is analyzed using linear regression (LR), and the MM elements can be reconstructed accordingly. Based on the analysis, this study gives corresponding suggestions for selecting appropriate polarimetric schemes to extract specific polarization properties, which can be helpful for the further development of quantitative tissue polarimetric imaging and biosensing.

## 2. Materials and Methods

### 2.1. Experimental Setup and Tissue Samples

**Figure 1:**
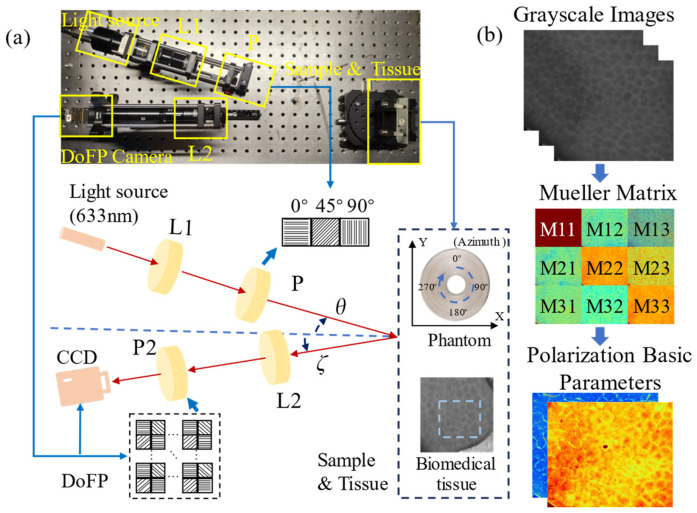
Schematics of the experimental setup and samples: (**a**) Backscattering MM setup using a DoFP camera. L1 and L2, lenses; P1 and P2, polarizers; *θ*, the oblique incidence angle; *ζ*, the oblique detection angle; sample and tissue, concentrically aligned silk phantom, and porcine liver tissue; (**b**) flowchart of polarization parameter acquisition based on the 3 × 3 MMs.

In this study, we adopt an experimental setup for 3 × 3 backscattering MM imaging based on a division of focal plane (DoFP) camera [29,30,31]. As shown in Figure 1a, the monochromatic light emitted from the LED (3 W, 633 nm, Δλ = 20 nm, Daheng Optic, Beijing, China) is modulated by the PSG consisting of a collimating objective lens L1 (Hengyang Optic, Guangzhou, China) and three fixed polarizers P1 (extinction ratio > 1000:1, LBTEK Optic, Changsha, China), and then scattered by a tissue sample. The P1 is driven by a screw linear motor M (FSK30, FUYU Technology, Chengdu, China) to generate 0°, 45°, and 90° linear states of polarization illuminations in series during the measurement. The scattered photons from the tissue samples are captured by the DoFP camera (PHX050S-P, Lucid Vision Labs, Richmond, BC, Canada) after passing through the imaging objective L2 (Hengyang Optic, Guangzhou, China). The DoFP camera contains numerous units capable of autonomously extracting intensity information from four different linear polarizers within different emergent lights. As illustrated in Figure 1b, by utilizing the grayscale images, we can construct the sample’s MMs and further analyze them by extracting the corresponding PBPs, which characterize the polarization properties of the tissue sample.

Here, we adopt an anisotropic scattering phantom consisting of concentrically aligned silk fibers, as shown in Figure 1a, which can generate the MMs of cylindrical scatterers along all azimuthal directions in the imaging X−Y plane in a single measurement [4]. Additionally, we utilize the porcine liver tissue as an ex vivo experimental sample for validation. This animal experimentation work was approved by the Ethics Committee of Tsinghua Shenzhen International Graduate School, Tsinghua University.

Prior to measurement, calibration of each optical component is conducted using a polarimeter (PAX1000, Thorlabs, Newton, NJ, USA) to ensure that systematic errors are maintained within 1%. During measurement, a constant distance between the PSA (or DoFP camera) and the tissue surface is rigorously maintained to minimize the errors arising from focal length variations. Meanwhile, the angles between the center line and both the PSG and sample arms are systematically adjusted to modulate *θ* and *ζ* as indicated in Figure 1a. The 3 × 3 MMs of the sample are reconstructed according to Equations (1) and (2), where *DoFP*, *L*_2_, *P*, and *L*_1_ correspond to the 3 × 3 MMs of the optical components, *S_in_* represents the input Stokes vector, and *S_out_* represents the output Stokes vector [13].

(1) $$S_{out}=DoFP\times L_{2}\times MM_{S}\times P\times L_{1}\times S_{in},$$

(2) $$MM_{S}=DoFP^{-1}\times S_{out}\times {S_{in}}^{-1}\times P^{-1}.$$

### 2.2. Azimuthal-Dependent Curves of Mueller Matrix Elements

**Figure 2:**
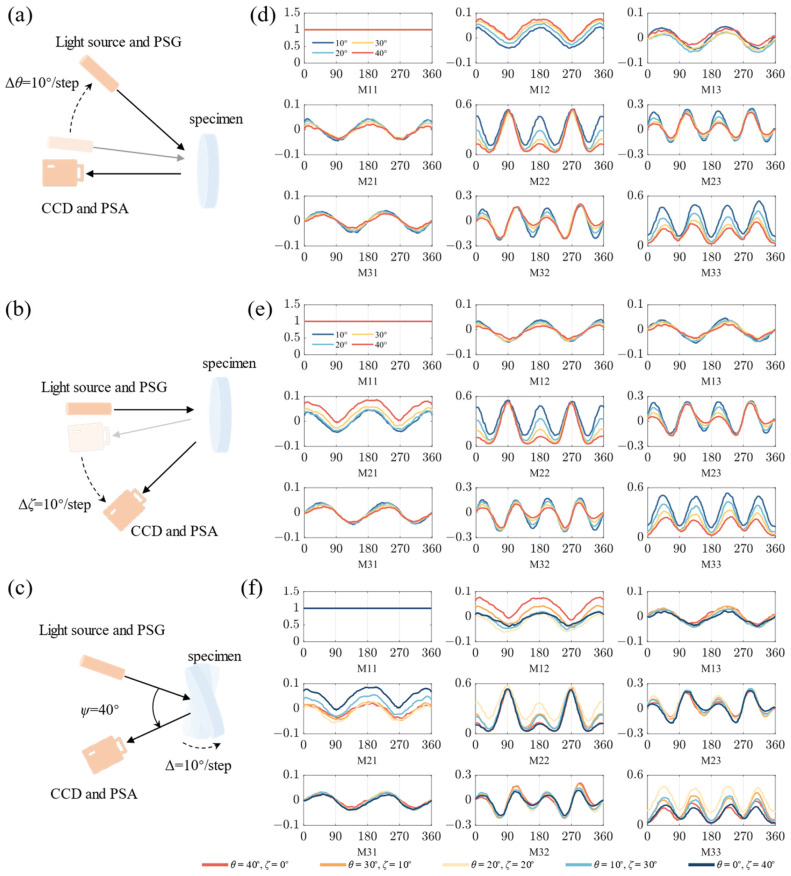
Azimuthal-dependent curves of 3 × 3 MM elements constructed as schemes (ii)−(iv): (**a**) incident angle *θ* modulated; (**b**) detection angle *ζ* modulated; (**c**) absolute spatial angle *ψ* modulated. The corresponding curves: (**d**) incident angle *θ* modulated; (**e**) detection angle *ζ* modulated; (**f**) absolute spatial angle *ψ* modulated. The horizontal axis shows the azimuth angle of the fibers, and the vertical axis represents the MM element values. All the elements are normalized by M11.

To comprehensively illustrate the aberrations in MM elements induced by complex spatial illumination, the azimuthal-dependent curves of nine MM elements are constructed according to the method described in our previous work [13]. As shown in Figure 2a,b, we maintain *ζ* (or *θ*) at 0° and systematically modulate *θ* (or *ζ*) from 0 to 40° in steps of 10°, respectively. Subsequently, the acquired curves are shown in Figure 2d,e. Upon comparing the two groups of curves, we can evidently notice that the curves of M12, M21, M13, and M31 exhibit opposite variations with increasing *θ* or *ζ*, while the curves of the other MM elements have identical variations with increasing *θ* or *ζ*. Additionally, as depicted in Figure 2f, we measure the azimuthal-dependent curves by simultaneously modifying both *θ* and *ζ*, shown in Figure 2c. These curves maintain the identical *ψ* and illustrate the influence of different distributions of *θ* and *ζ* on the aberrations. A detailed analysis will be provided in Section 3.

### 2.3. Analysis Methods

For quantitative analysis, we define the MMs measured when *ψ* is 10° and *θ* or *ζ* are equal to 0° as the referenced MMs. The MSEs between the curves for the other *θ* or *ζ* and the corresponding reference curves are calculated as Equation (3) for the two-periodic elements M12, M21, M13, and M31, respectively. In Equation (3), *C*(*M_j_*) represents the calculated azimuthal-dependent curve, *C*(*M_ref_*) represents the corresponding reference curve, and *N* represents the point number in the curves. In Figure 2d,e, the M12 and M21 curves exhibit a noticeable shift along the vertical axis. Thus, we calculate the direct current components of energy spectral density for different curves.

(3) $$MSE(C(M_{j}),\;C(M_{ref}))=\frac{1}{N}\sum_{i=1}^{N}{\left\|C{\left(M_{j}\right)}_{i}-C{\left(M_{ref}\right)}_{i}\right\|}^{2}.$$

Since the anisotropic silk fiber phantom is circularly shaped, we introduce the ellipticity as a parameter to characterize the image distortions induced by the increasing *ζ*, as shown in Equation (4), where *y* and *x* are the longitudinal and transverse distances of the phantom and *r* is the radius of the phantom. The analytical relationship between *ζ* and the ellipticity is also shown in Equation (4).

(4) $$Ellipticity=\frac{y-x}{y}=\frac{r-r\cos\varsigma }{r}=1-\cos\varsigma .$$

The ellipticity results are analyzed through the LR and least squares method in order to validate the correctness of the analytical relationship. In Equations (5) and (6), *x* is the deg of *θ* or *ζ*, *y* is the ellipticity of corresponding *x*, $\bar{x}$ and $\bar{y}$ are the means of *x* and *y*, and *n* is the data number.

(5) $$Variable=\frac{\sum xy-n\bar{x}\bar{y}}{\sum x^{2}-n{\bar{x}}^{2}},$$

(6) $$Intercept=\bar{y}-Variable\cdot \bar{x}.$$

To validate the significant alleviation of aberrations in PBPs achieved by measuring MMs with an adjusting distribution of *θ* and *ζ*, we characterize the depolarization of the tissue sample by measuring the *LDoP* (linear degree of polarization) and *b*_2_ (linear depolarization and anisotropies) parameters [32], which are sensitive to the oblique incidence [13,33]. They can be calculated as Equations (7)–(9).

(7) $$b=\frac{M22+M33}{2},$$

(8) $$b_{2}=1-b,$$

(9) $$LDoP=\frac{M21+M22}{M11+M12}.$$

We construct the frequency distribution histograms (FDHs) of the *LDoP* and *b*_2_ parameters, which can systematically characterize the distribution of the overall polarization properties with PBP images. Several image distance measurements, such as the histogram distance (*HD*), Euclidean norm, and Structure Similarity Index Measure (*SSIM*) [34], are utilized to evaluate the alleviation effects quantitatively. Meanwhile, these measurements can also be used to identify the structural information as different schemes. The formulas for these measurements can be described as Equations (10) and (11),

(10) HD(x,y)=MSE(histogram(x),histogram(y)),

(11) $$SSIM(x,y)=\frac{\left(2\mu_{x}\mu_{y}+C_{1})(2\sigma_{xy}+C_{2}\right)}{\left(\mu_{x}^{2}+\mu_{y}^{2}+C_{1})(\sigma_{x}^{2}+\sigma_{y}^{2}+C_{2}\right)},s.t.C_{1}={\left(0.01\times L\right)}^{2},\;C_{2}={\left(0.03\times L\right)}^{2},$$

where *μ_x_*, *μ_y_*, *σ_x_*, *σ_y_*, and *σ_xy_* are the local means, standard deviations, and cross-covariance for MM images *x*, *y*, and *L* = 1 for MM images.

## 3. Results

### 3.1. Aberrations Induced by Complex Spatial Illumination of Two-Periodic MM Elements

It is widely recognized that the *D_L_* derived from M12 and M13 represents the linear diattenuation, while the *P_L_* derived from M21 and M31 represents the linear polarizance of the tissue sample [32]. *D_L_* and *P_L_* can be calculated as Equations (12) and (13).

(12) $$D_{L}=\sqrt{M12^{2}+M13^{2}},$$

(13) $$P_{L}=\sqrt{M21^{2}+M31^{2}}.$$

**Figure 3:**
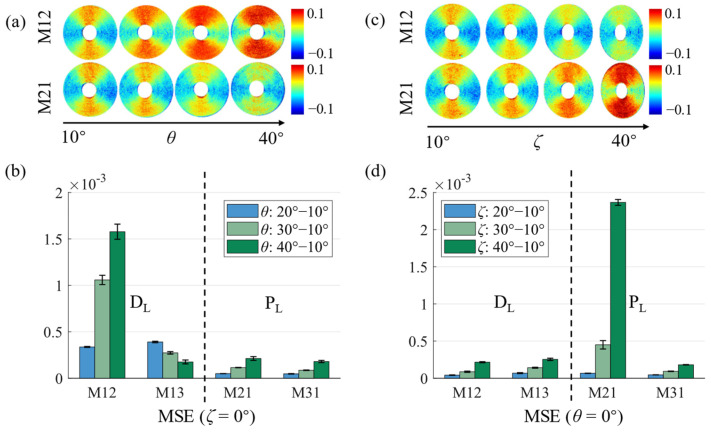
M12 and M21 images and analysis of anisotropic silk phantom under different *θ* and *ζ*: (**a**) M12 and M21 images with *θ* from 10° to 40°; (**b**) MSE with different *θ*; (**c**) M12 and M21 images with *ζ* from 10° to 40°; (**d**) MSE with different *ζ*.

Previously, we demonstrated that the symmetries of the M12 and M21 pairs, together with the M13 and M31 pairs, can be broken when *θ* is more than 20° [13]. In this work, we measure MMs and construct the azimuthal-dependent curves of the anisotropic silk phantom, as in schemes (ii) and (iii), as shown in Figure 2d and Figure 2e, respectively. The azimuthal-dependent curve values of M12, M21, M13, and M31 are within the range of -0.1 and 0.1 when *θ* and *ζ* are less than 20°. As *θ* increases, the M12 curve progressively shifts towards positive, while the M21 and M31 curves exhibit amplitude variations but maintain both positive and negative values. Similarly, as *ζ* increases, the M21 curve shifts towards positive, while the M12 and M13 curves have variations in amplitude but still exist as positive and negative values. Furthermore, these can be more clearly seen in the MM images of the phantom in Figure 3a,c. It should be noted that the curves’ variations of other MM elements are identical, as in both schemes (ii) and (iii), like period degeneracy in four-periodic elements. These observations imply that the different polarimetric aberrations in two-periodic elements with identical *ψ* may be directly correlated to different schemes.

In Figure 3b,d, we quantitatively analyze difference aberrations in MM elements by calculating the MSE of curves using schemes (ii) and (iii), respectively. Different ring areas with various radii for each MM element are collected to calculate the means and standard deviations (SDs) of the MSE. The error bars on the plots represent the SDs of the data. In consideration of the image distortions due to the oblique emergent light, the SDs, which do not exceed 5% of the means, validate the reliability of our experimental results. As shown in Figure 3b,d, there is a significant difference between the two conditions, as in schemes (ii) and (iii). Figure 3b shows significant increases in the MSE between the M12 curves with *θ* exceeding 20° and the referenced M12 curve. Notably, when *θ* increases to 40°, the MSE even rises to 1.5 × 10^−3^, indicating a significant aberration in M12. Similarly, Figure 3d shows significant increases in the MSE between the M21 curves with *ζ* exceeding 20° and the referenced M21 curve. Notably, when *ζ* increases to 40°, the MSE even rises to 2.3 × 10^−3^, indicating a significant aberration in M21. Additionally, the curves of M12, M13, M21, and M31 are similar to the trigonometric functions, and the curves of M12 and M21 shift along the vertical axis with an increase in *θ* or *ζ*. Therefore, we calculate the direct current components of the energy spectral density at the above eight different angles to quantitatively analyze the aberrations, as shown in Table 1. For most curves, their direct current components tend to be zero, and the variations do not exceed 3 × 10^−4^, excluding the curves of M12 or the curves of M21. When *θ* or *ζ* attain significant magnitudes, such as achieving 40°, the direct current components of the M12 and M21 curves can escalate to 2 × 10^−3^. These results also show that false-positive linear diattenuation is induced as *θ* increases and false-positive linear polarizance is induced as *ζ* increases. Through these quantitative analyses, it can be proven that the aberrations are different from schemes (ii) and (iii), which cannot be ignored in the polarization properties analysis.

In summary, when *θ* or *ζ* is beyond 30°, we can obtain more accurate M21 and M31, as in scheme (ii), or obtain more accurate M12 and M13, as in scheme (iii); however, it should be noted that image distortions will be included by the oblique emergent light.

### 3.2. MM Image Distortions Induced by Oblique Emergent Light

**Figure 4:**
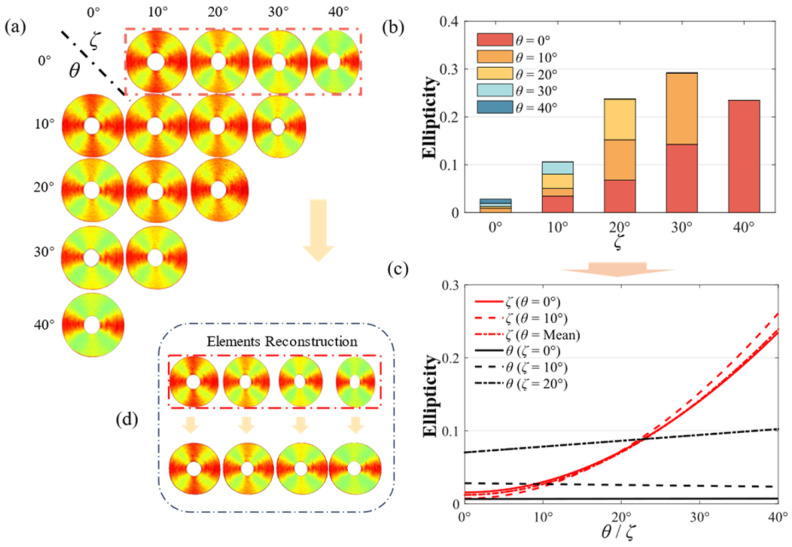
Schematic of ellipticity variation: (**a**) the M22 elements for different *θ* and *ζ*; (**b**) histogram of ellipticity for different *θ* and *ζ*; (**c**) LR fitting curves obtained by the ellipticity data; (**d**) the reconstruction of the MM elements.

We find that the MM images of the phantom undergo transverse compressions, defining the transverse direction as parallel to the incident plane. The transverse compressions result in the MM image distortions and the destruction of their structural information. To quantitatively analyze the distortions, ellipticity is utilized as a characterization parameter, which represents the distortions of the MM images and can be obtained by Equation (4). As shown in Figure 4a, it is evident that the variations in *θ* exert minimal influence on the distortions of the MM images, while the shape of the MM images transforms into an ellipse with an increasing *ζ*. In Figure 4b, the lengths of the columns with the identical color, representing the identical *θ*, progressively escalate as *ζ* increases from 10° to 40°. Notably, this phenomenon is most obvious when *θ* is 0° or 10°. This intuitively illustrates that the increase in *ζ* aggravates the extent of distortions, while the varying *θ* has no obvious influence on it.

Therefore, according to the experimental setup model, the analytical relationship between ellipticity and *ζ* is constructed as shown in Equation (4). The analytical relationship illustrates that the cosine value of *ζ* has a linear relationship with the ellipticity of the MM images. As shown in Figure 4c, we utilize the LR to verify the correctness of the analytical relationship using the above ellipticity data. The fitting curves for the variable *θ* and the constant *ζ* are predominantly close to horizontal, indicating a limited association between ellipticity and *θ*. Additionally, these curves possess different intercepts, indicating that *ζ* has a predominant influence on ellipticity. Conversely, the curves for the constant *θ* and the variable *ζ* are extremely aligned with the curves constructed in Equation (4), affirming the accuracy of the analytical relationship. Moreover, we can introduce a compensation coefficient *σ* to mitigate the distortions in the transverse direction through Equation (14), where *x_mit_* is the distance of the reconstructed MMs and *x_dis_* is the distance of the distorted MMs. The reconstructed MM images can be obtained using the bilinear interpolation algorithm. As shown in Figure 4d, this approach effectively mitigates distortions in the MM images.

(14) $$x_{mit}=\sigma x_{dis},\;\sigma =\frac{1}{1-Ellipticity}.$$

To systematically verify the reliability of our conclusions, the calculated results of LR based on the least squares method are demonstrated in Table 2. When *θ* is treated as the variable, the slope of the regression equation converges to approximately zero, while the intercept enlarges with an increasing *ζ*. Conversely, when *ζ* is considered as the variable, the slope and the intercept are approximately equal to −1 and 1, aligning closely with the values outlined in Equation (4), respectively. The conspicuous linear correlation between *ζ* and ellipticity leads to an exceedingly small F below 0.5%, indicating a statistically significant correlation. The exceedingly small *p*-value, which is also below 0.5%, further reinforces the reliability of the above conclusions. All of these conclusions are applicable to the majority of distortion models caused by oblique emergent light and can be used for MMs’ reconstruction, which can recover their structural information. Consequently, we advocate for obtaining MM measurements using scheme (ii) rather than scheme (iii) to acquire undistorted MM images. In instances where measuring MMs using scheme (iii) is deemed indispensable, we suggest restoring the structural integrity of the images using Equation (14).

### 3.3. Calibration of Polarization Properties by Adjusting the Distribution of θ and ζ

It should be noted that MMs measured using schemes (ii) or (iii) with *ψ* exceeding 20° display significant aberrations, which could potentially induce inaccuracies in characterizing the polarization properties. However, we find that measuring MMs with an adjusting distribution of *θ* and *ζ*, as in scheme iv), can remarkably alleviate the aberrations. In Figure 2f, by observing the azimuth dependence curve with *θ* equal to *ζ*, some qualitative variations are revealed, as follows: (1) The vertical displacements between the M12, M21, M13, and M31 curves and referenced curves are maintained within 0.03. (2) The M22 curve has a sharp peak at the special azimuth location of 90°. (3) Both the M23 and M32 curves have two sharp peaks at the special azimuth locations of 150° and 210°, respectively. (4) The M33 curve amplitude and minimum approximately increase to 0.4 and 0.2, respectively. These variations make the MMs’ curves extremely congruent with the referenced curves. Meanwhile, period degeneracy occurs in the central-block MM elements when *θ* or *ζ* exceed 20°, as in schemes (ii) and (iii), which achieves excellent periodic recovery when *θ* approaches approximate equality to *ζ*, as in scheme (iv), as shown in Figure 4a. These results reveal that the MMs measured with an adjusting distribution of *θ* and *ζ* exhibit a great resemblance to the referenced MMs, which may provide a strategy for obtaining accurate MMs with *ψ* exceeding 30°.

**Figure 5:**
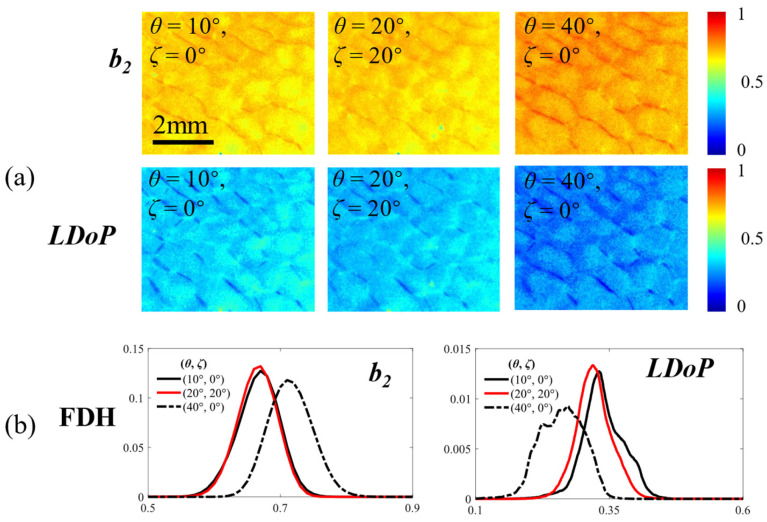
Polarization property recovery: (**a**) parameter images of *LDoP* and *b*_2_ at different *θ* and *ζ*; (**b**) corresponding FDHs of the curves.

To further explore the improvement of the polarization properties through this strategy, we measure MMs on porcine liver tissue to validate the alleviation effect on the aberrations in *LDoP* and *b*_2_. To avoid the destruction of the structural information, we adopt scheme (ii) to measure MMs when *ζ* is 0°. As depicted in Figure 5a, subtle structural distortions are observed in the measured MM images when *θ* and *ζ* are 20°. Notably, the polarization properties of the microstructure, including *LDoP* and *b*_2_, are perceived to be more accurate when using scheme (iv). When *θ* is 40° and *ζ* is 0°, the aberrations of the PBPs validate the false-positive and false-negative calculated results of depolarization. It should be noted that *b*_2_ is commonly utilized in cancer detection due to its sensitivity to small particles, so the incidence of the above phenomena needs to be reduced [32]. Furthermore, in Figure 5b, when *θ* is 40° and *ζ* is 0°, the curves exhibit substantial peak shifts along the horizontal axis compared to the referenced curves, which can be recovered by measurement with an adjusting distribution of *θ* and *ζ*. We calculate *HD*, where a smaller distance indicates a higher precision in polarization properties, to verify the reliability of the conclusion. The tabulated results reveal that parameters *LDoP* and *b*_2_ with *θ* and *ζ* equal to 20° are closer to the corresponding results of the reference parameters, which merely demonstrate 1.4% and 12% *HD*s in comparison to the parameters measured in scheme (ii). These results illustrate the capability of measuring MMs using scheme (iv) to preserve nearly all accurate depolarization information. Furthermore, measuring polarization properties using scheme (iv) can be more accurate and avoid uncertain aberrations caused by oblique incidence or oblique emergent light in biological tissues.

To assess the precision of the recovery, we calculate the Euclidean norm and *SSIM* between the PBP images using scheme (ii) (or scheme (iv)) and the referenced PBP images. In Table 3, employing the Euclidean norm as a metric reveals that the distances between the *b*_2_ and *LDoP* images using scheme (iv) and the referenced images are only 11.53 and 19.96, respectively, indicating an effective overall recovery. Meanwhile, despite the MM images being distorted, as in scheme (iv), the higher *SSIM* values of *b*_2_ and *LDoP*, which imply greater similarity to the original structure, suggest that the precision of the polarization properties in the microstructure can partly compensate for the loss of macroscopic structural information. Therefore, when *ψ* exceeds 30°, measuring MMs with an adjusting distribution of *θ* and *ζ* is more precise if the overall MMs and PBPs are of the utmost concern.

## 4. Discussion

MM polarimetry plays a crucial role in optical biosensors due to its significant performance and advantages in mapping the microscopic morphological information of biological tissues. In our previous work, we focused on the influence of oblique incidence with normal emergent light, as in scheme (ii), and proposed specific optimizing strategies [13]. Here, we further explore the detailed characteristics of aberrations induced in all non-collinear conditions, as in schemes (ii)–(iv), which are adaptable to more scenarios in practical polarimetric applications. The conclusions of this study for scheme (ii) are consistent with our previous work, while the analysis and optimization strategies of schemes (iii) and (iv) are the essential aims of this work, providing a thorough complement to the previous conclusions. Furthermore, this study serves as a methodological contribution to offer valuable insights into the design and construction of optical systems for backscattering MM polarimetry in bioimaging and sensing applications, such as bio-structural and optical properties sensing [14,15], polarimetric endoscopy [16,17], and skin tissue evaluation [18,19,20].

## 5. Conclusions

In this study, we systematically analyzed the polarimetric aberrations induced by three different illumination schemes in backscattering MM imaging. These schemes were distinguished by incidence and emergent light in non-collinear conditions. Additionally, comprehensive comparisons were performed among the MMs obtained under these schemes, considering *ψ* as 10°, 20°, 30°, and 40°, respectively. We found that measuring MMs with oblique emergent light can induce specific distortion in MM images, hindering the characterization of tissue structural information. Notably, a linear relationship emerged between the cosine value of *ζ* and the extent of transverse compression, and this could be used to reconstruct the MM images. Additionally, when measuring MMs as in scheme (ii) with *θ* exceeding 20°, the aberrations of M12 and M13 exhibited increasing intensities. When it came to scheme (iii) with *ζ* exceeding 20°, M21 and M31 exhibited significantly increasing intensities. However, the variations in other MM elements remained identical. It should be noted that measuring MMs as different schemes may have different effects on the polarization parameters. Furthermore, we found that scheme (iv) could alleviate the polarimetric aberrations. Quantitative analysis of PBPs using porcine liver tissues revealed a significant improvement in the alleviation of PBP aberrations when MMs were measured with an adjusting distribution of *θ* and *ζ*. These findings delivered some crucial guidance for choosing the appropriate spatial illumination for non-collinear MM imaging, as follows: (1) If possible, measure MMs with a normal emergent light to obtain accurate structural information. (2) For linear polarizance, measure MMs as in scheme (ii) to accurately obtain M21 and M31. (3) For linear diattenuation, measure MMs as in scheme (iii) to obtain M12 and M13 accurately. (4) To obtain overall MMs and PBPs, measure MMs as in scheme (iv) with an adjusting distribution of *θ* and *ζ*. In summary, the optimized schemes provided critical criteria for the spatial illumination scheme selection of non-collinear backscattering MM measurements, which can be helpful for the further development of quantitative tissue polarimetric imaging and biosensing.

## Figures and Tables

**Table 1:** Direct current components of the energy spectral density with different MM elements.

Degrees	$M12\;(\times 10^{-5})$	$M13\;(\times 10^{-5})$	$M21\;(\times 10^{-5})$	$M31\;(\times 10^{-5})$
*θ* = 10°, *ζ* = 0°	0.87	0.38	0.30	0.13
*θ* = 20°, *ζ* = 0°	40.83	25.66	0.29	0.0080
*θ* = 30°, *ζ* = 0°	119.83	12.61	0.07	0.03
*θ* = 40°, *ζ* = 0°	173.45	2.49	2.42	0.01
*θ* = 0°, *ζ* = 10°	0.89	1.04	0.68	0.10
*θ* = 0°, *ζ* = 20°	3.11	2.98	4.27	0.19
*θ* = 0°, *ζ* = 30°	4.13	0.29	48.64	1.13
*θ* = 0°, *ζ* = 40°	5.87	3.81	252.78	4.24

**Table 2:** LR results.

Different Degrees	Intercept	X Variable	Significance F	*p*-Value
*θ* (*ζ* = 0°)	0.0066	0.000013	0.92	0.21
*θ* (*ζ* = 10°)	0.028	−0.00012	0.80	0.070
*θ* (*ζ* = 20°)	0.070	0.00080	0.30	0.052
*ζ* (*θ* = 0°)	0.95	−0.93	0.0014	0.0011
*ζ* (*θ* = 10°)	1.091	−1.08	0.0058	0.0051
*ζ* (*θ* = Mean)	0.98	−0.96	0.000054	0.000040

**Table 3:** Quantitative results of the recovery by different similarity metrics.

Similarity Metrics	*b*_2_ (*θ*, *ζ*)		*LDoP* (*θ*, *ζ*)	
	(20°, 20°)	(40°, 0°)	(20°, 20°)	(40°, 0°)
*HD* (×10^−6^)	0.16	10.83	1.29	10.67
Euclidean norm	11.53	49.67	19.96	79.15
*SSIM*	0.90	0.88	0.81	0.75

## Data Availability

Data underlying the results presented in this paper are not publicly available at this time but may be obtained from the authors upon reasonable request.
